# Supplementary material for: Predicting visual function by interpreting a neuronal wiring diagram
Source: Nature. 2024 Oct 2;634(8032):113–23. doi: 10.1038/s41586-024-07953-5 (PMC11446822; doi:10.1038/s41586-024-07953-5)
Supplement: Supplementary file 6 — CRF and ERF predictions for individual TmY4 and TmY9 cells. Analogous to Supplementary Data 3, but for TmY target types. Shown are the top four monosynaptic pathways, the strongest pathway passing through each of the top ten intermediary types (ranking from Extended Data Fig. 7), and the trisynaptic pathway Tm1–TmY–Dm3–TmY (see the section entitled Prediction of spatial normalization). [file 41586_2024_7953_MOESM6_ESM.zip › DataS4/TmY9qΓèÑ/720575940613386386.pdf]

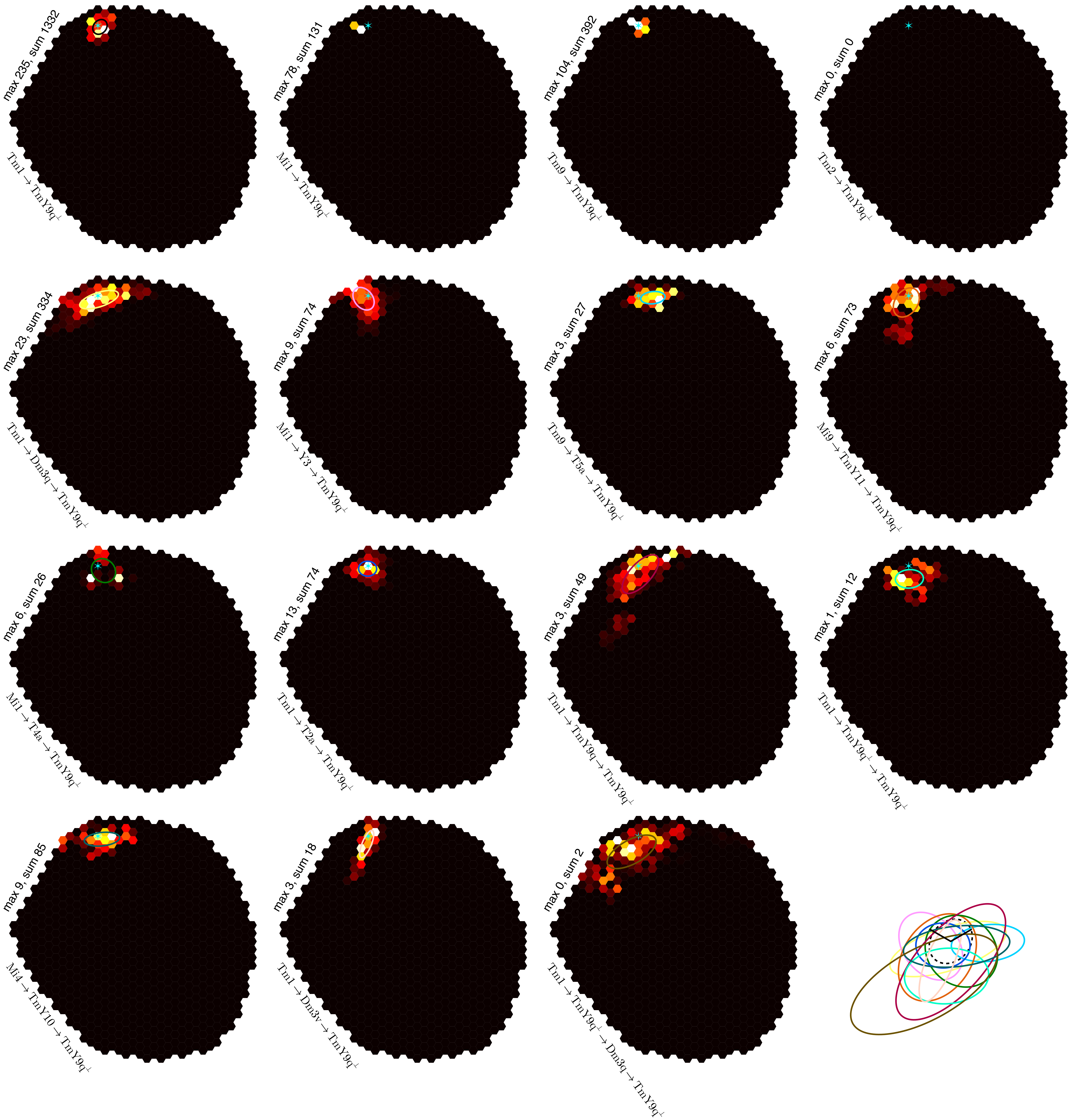

max 235, sum 1332
Tm1 → TmY9q⊥
max 78, sum 131
Mi1 → TmY9q⊥
max 104, sum 392
Tm9 → TmY9q⊥
max 0, sum 0
Tm2 → TmY9q⊥
max 23, sum 334
Tm1 → Dm3q → TmY9q⊥
max 9, sum 74
Mi1 → Y3 → TmY9q⊥
max 3, sum 27
Tm9 → T5a → TmY9q⊥
max 6, sum 73
Mi9 → TmY11 → TmY9q⊥
max 6, sum 26
Mi1 → T4a → TmY9q⊥
max 13, sum 74
Tm1 → T2a → TmY9q⊥
max 3, sum 49
Tm1 → TmY9q → TmY9q⊥
max 1, sum 12
Tm1 → TmY9q⊥ → TmY9q⊥
max 9, sum 85
Mi4 → TmY10 → TmY9q⊥
max 3, sum 18
Tm1 → Dm3v → TmY9q⊥
max 0, sum 2
Tm1 → TmY9q⊥ → Dm3q → TmY9q⊥
